# Forecasting the monthly incidence of scarlet fever in Chongqing, China using the SARIMA model

**DOI:** 10.1017/S0950268822000693

**Published:** 2022-04-21

**Authors:** W. W. Wu, Q. Li, D. C. Tian, H. Zhao, Y. Xia, Y. Xiong, K. Su, W. G. Tang, X. Chen, J. Wang, L. Qi

**Affiliations:** 1 Chongqing Center of Disease Control and Prevention, Chongqing 400042, China; 2 School of Public Health (Shenzhen), Sun Yat-sen University, Shenzhen, Guangdong 510275, China

**Keywords:** Forecasting, infectious disease, SARIMA model, scarlet fever, time series analysis

## Abstract

The incidence of scarlet fever has increased dramatically in recent years in Chongqing, China, but there has no effective method to forecast it. This study aimed to develop a forecasting model of the incidence of scarlet fever using a seasonal autoregressive integrated moving average (SARIMA) model. Monthly scarlet fever data between 2011 and 2019 in Chongqing, China were retrieved from the Notifiable Infectious Disease Surveillance System. From 2011 to 2019, a total of 5073 scarlet fever cases were reported in Chongqing, the male-to-female ratio was 1.44:1, children aged 3–9 years old accounted for 81.86% of the cases, while 42.70 and 42.58% of the reported cases were students and kindergarten children, respectively. The data from 2011 to 2018 were used to fit a SARIMA model and data in 2019 were used to validate the model. The normalised Bayesian information criterion (BIC), the coefficient of determination (*R*^2^) and the root mean squared error (RMSE) were used to evaluate the goodness-of-fit of the fitted model. The optimal SARIMA model was identified as (3, 1, 3) (3, 1, 0)_12_. The RMSE and mean absolute per cent error (MAPE) were used to assess the accuracy of the model. The RMSE and MAPE of the predicted values were 19.40 and 0.25 respectively, indicating that the predicted values matched the observed values reasonably well. Taken together, the SARIMA model could be employed to forecast scarlet fever incidence trend, providing support for scarlet fever control and prevention.

## Introduction

Scarlet fever is a contagious disease caused by group A *streptococcus pyogenes* (GAS), primarily affecting school children aged between 2 and 10 years [1]. Patients and scarlet fever streptococcus carriers are the main infectious sources of scarlet fever. GAS is transmitted from human to human via direct contact, droplet spread from people with pharyngeal colonisation or carriage and contaminated fomites in addition to being reportedly food borne [2]. The incubation period of scarlet fever is 2 to 10 days, and transmission period usually lasts 10 to 21 days without appropriate treatment [3]. The symptoms of the disease include cute fever, sore throat, and diffuse bright red rash and obvious desquamation after rash regression [4]. The incidence rate of scarlet fever is high in kindergartens and primary schools, but extremely low in children under the age of 2 because they are protected by maternal antibodies [5]. Scarlet fever has been a feared killer during the eighteenth and nineteenth centuries in Europe, but it disappeared in twentieth century because of widely use of antibiotics as well as advancements in health care and nutrition [6]. However, in past decade, scarlet fever had resurged in several countries and districts, including Hong Kong and South Korea in Asia, and UK in Europe [7–9]. The causes of resurgence of scarlet fever may include microbial, host and meteorological factors [10]. Due to lack of vaccines for streptococcus pyogenes infection, the resurgence of scarlet fever has become a public health problem of global concern [11].

Accurately modelling and forecasting the scarlet fever epidemic holds great importance as it helps the authorities make the necessary arrangements and allows a timely response. Many statistical models have been used for forecasting and early warning of disease outbreaks. One of the most popular model is the autoregressive integrated moving average model (ARIMA), which has been used to describe the trends of many diseases in epidemiological studies, including Haemorrhagic fever, H5N1 avian influenza and COVID-19 [12–14]. Seasonal autoregressive integrated moving average (SARIMA) model considers the overall trend as well as seasonal variation, which was widely used in time series modelling [15]. Since scarlet fever presents typical seasonal characteristics, a SARIMA model may be more effective than ARIMA model in predicting the scarlet fever epidemic. However, no research has focused on the prediction of scarlet fever epidemics that considered specific seasonal trends observed in Chongqing, Southwest China. Therefore, in this study, we aimed to develop a prediction model using SARIMA model for scarlet fever in Chongqing, China.

## Material and methods

### Study region

**Fig. 1.**
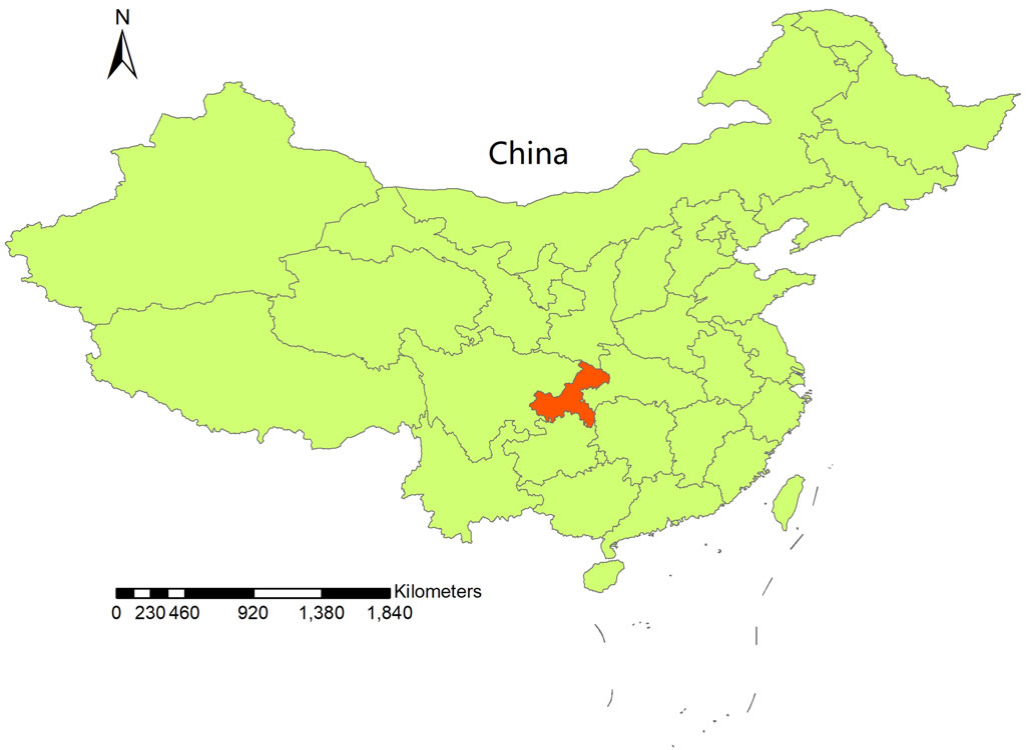
Geographical location of Chongqing, China.

With the latitude of 28°10′~32°13′ and longitude of 89°35′~103°04′, Chongqing is the largest municipality with over 32 million registered inhabitants in Southwest of mainland China (Fig. 1). The city of Chongqing has a subtropical monsoon humid climate characterised by a hot, humid summer and a cold, dry winter. The annual average temperature and precipitation in the city are 17°C and 1100 mm, respectively.

### Data collection

All scarlet fever cases diagnosed in hospitals were reported to ‘National Disease Reporting Information System’ (NDRIS) within 24 h after diagnoses since 2004, according to the requirement of the law of the People's Republic of China on Prevention and Treatment of Infectious Diseases. The scarlet fever cases included clinical diagnosed and laboratory-confirmed cases. The diagnosis criteria for scarlet fever were provided by National Health Commission of the People’ Republic of China in 2008. A clinical diagnosed scarlet fever case was defined as a patient with fever, angina, rash, which rapid detection of group A *Streptococcus* test positive. A laboratory-confirmed case must be based on an aetiological examination. In Chongqing, scarlet fever can only be diagnosed in hospitals authorised by the health commission. We retrieved monthly reported scarlet fever cases information during January 2011 to December 2019 from NDRIS, including basic characteristics and the date of symptoms onset of cases. Additionally, the map of Chongqing was downloaded from National Earth System Science Data Center (http://www.geodata.cn/).

### SARIMA model construction

The classical ARIMA (*p*, *d*, *q*) model is a frequently used technique for forecasting time series data. It has been proved as an effective and useful forecasting tool [16]. The SARIMA model is an extension of ARIMA model, which contains seasonal characteristics of time series [17]. The basic form of a SARIMA model is represented as SARIMA (*p*, *d*, *q*) (*P*, *D*, *Q*) *s*, where AR is the autoregressive model and MA is the moving average model; *d* and *D* represent the non-seasonal and seasonal differencing, respectively; *p*, *q*, *P* and *Q* denotes the orders of non-seasonal and seasonal autoregressive and moving average order, respectively; *s* denotes the length of seasonal period of the observed series, defined as 12 [18]. The formula of the SARIMA (*p*, *d*, *q*) (*P*, *D*, *Q*) *s* model is:
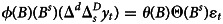

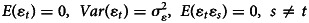

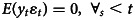

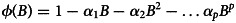

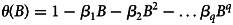

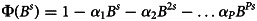

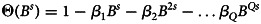


Where *B* denotes the backward shift operator, *y*_*t*_ represents the actual number of scarlet fever cases at time *t*, and *ɛ*_*t*_ denotes the residual errors from the SARIMA model. *ϕ*(*B*) is the non-seasonal *p* order autoregressive coefficient polynomial, and *θ*(*B*) is the non-seasonal *q* order autoregressive coefficient polynomial. 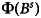 denotes the seasonal *P* order autoregressive coefficient polynomial, Θ (*B*^*s*^) denotes the seasonal *Q* order autoregressive coefficient polynomial.

The entire time series data was divided into two parts: a training set consisting of data collected from 1 January 2011 to 31 December 2018 to fit the models, and a validation set collected from 1 January to 31 December 2019 to validate the fitted models. The process of the SARIMA model involves four steps [18]: Firstly, a time series graph of monthly scarlet fever cases from 2011 to 2019 was drawn to test its stationary intuitively. Then, non-seasonal difference or seasonal difference should be used to transform the time series to achieve stationarity if the original data is not stationary. The stationarity was checked by the autocorrelation function (ACF) and partial autocorrelation function (PACF) of the differenced series. Secondly, the orders of *p, q, P* and *Q* were identified by using the ACF and PACF. The least square method was used to estimate the model parameters. Thirdly, the Bayesian information criterion (BIC), the coefficient of determination (*R*^2^) and the root mean squared error (RMSE) were used to evaluate the goodness-of-fit of the SARIMA models. The model with the lowest BIC and RMSE values, the highest *R*^2^ value was regarded as the optimal model. The Ljung-Box *Q* test, ACF and PACF of the residuals were used to diagnose whether the residuals sequence was a white noise sequence. Finally, the optimal model was used for a prospective prediction, and the RMSE and mean absolute per cent error (MAPE) were used to assess the accuracy of the model.

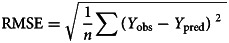


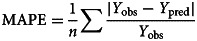


Where *Y*_obs_ and *Y*_pred_ represent the observed and predicted numbers of scarlet fever cases, respectively, and *n* represents the number of months for forecasting.

The statistical analysis was performed with SPSS 22.0 (SPSS Inc., USA), and the map was created with ArcGIS software (version 10.2, ESRI Inc., Redlands, CA, USA). *P* < 0.05 was considered as statistical significance.

## Results

### Descriptive analysis

A total of 5073 scarlet fever cases were reported in Chongqing from 2011 to 2019, with an average annual incidence of 1.87 per 100 000 (ranged from 1.16 per 100 000 to 2.72 per 100 000). The male to female ratio was 1.44, with 2997 male cases and 2076 female cases. The median age of the cases was 6 years old and 81.86% were children aged 3 to 9 years old. Regarding to the population classification, students have the highest proportion (42.70%, 2166), followed by children in kindergarten (42.58%, 2160) and scattered children (13.50%, 685) (Table 1).

**Table 1.** The demographic characteristics of scarlet fever cases in Chongqing, 2011–2019

Year	2011	2012	2013	2014	2015	2016	2017	2018	2019	Total
Gender										
Male	287	269	197	232	493	295	312	421	491	2997
Female	169	178	144	169	323	189	242	313	349	2076
Sex ratio	1.70	1.51	1.37	1.37	1.53	1.56	1.29	1.35	1.41	1.44
Age										
0–2	42	45	39	44	60	46	45	63	50	434
3–9	340	326	274	315	683	398	470	631	716	4153
10–14	48	64	21	38	53	32	33	33	58	380
≥15	26	12	7	4	20	8	6	7	16	106
Occupation										
Scattered children^a^	74	75	69	57	92	66	69	106	77	685
Kindergarten Children	157	153	137	160	375	234	255	329	360	2160
Students	206	214	131	181	337	179	225	295	398	2166
Others	19	5	4	3	14	5	5	4	5	62

a Scattered children refer to children who do not reach the age of 3 years old to go kindergarten, or are taken care of by their family members.

### Temporal pattern

**Fig. 2.**
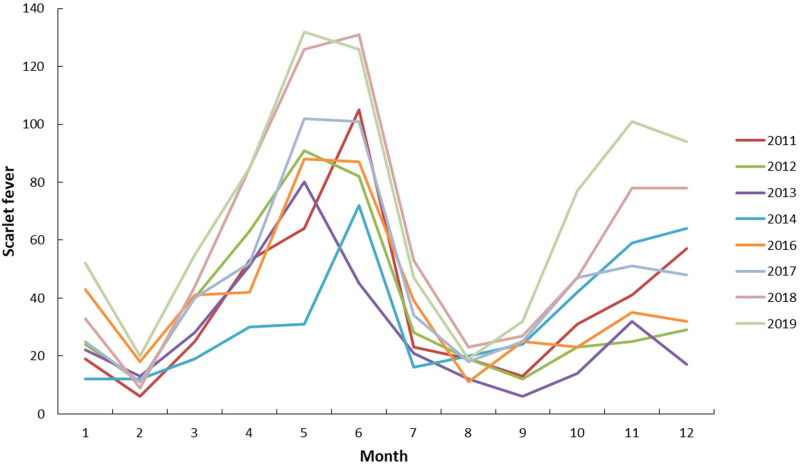
Monthly scarlet fever cases from 2011 to 2019 in Chongqing, China.

**Fig. 3.**
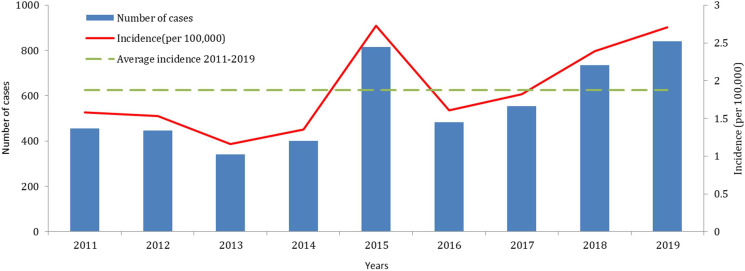
Annual incidence and number of scarlet fever cases reported in Chongqing, 2011–2019.

**Fig. 4.**
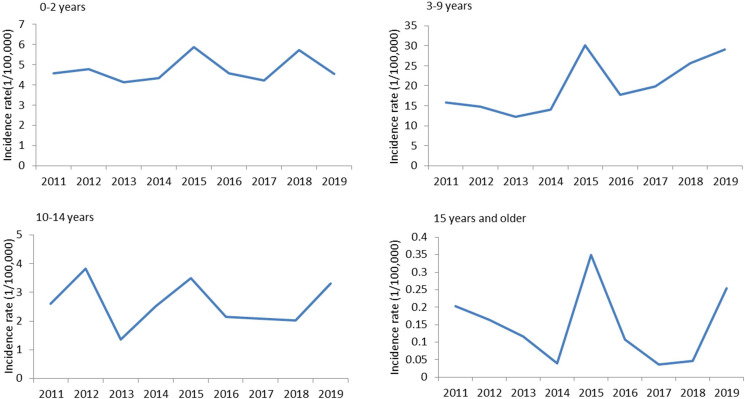
The incidence of scarlet fever by age group in Chongqing.

The variation of yearly and monthly distributions of scarlet fever cases was shown in Figures 2 and 3. Semiannual peaks of scarlet fever were observed during the study period, with peaks occurred from April to June and October to December each year (Fig. 2). The first peaks were much higher than the second peaks. The incidence of scarlet fever was highest in 2015 (2.72 per 100 000) (Fig. 3). Figure 4 showed that the annual incidence rate among children aged 3–9 years was increasing gradually, with the incidence rate peaked in 2015. The incidence rates of other age groups remain stable.

### Spatial pattern

**Fig. 5.**
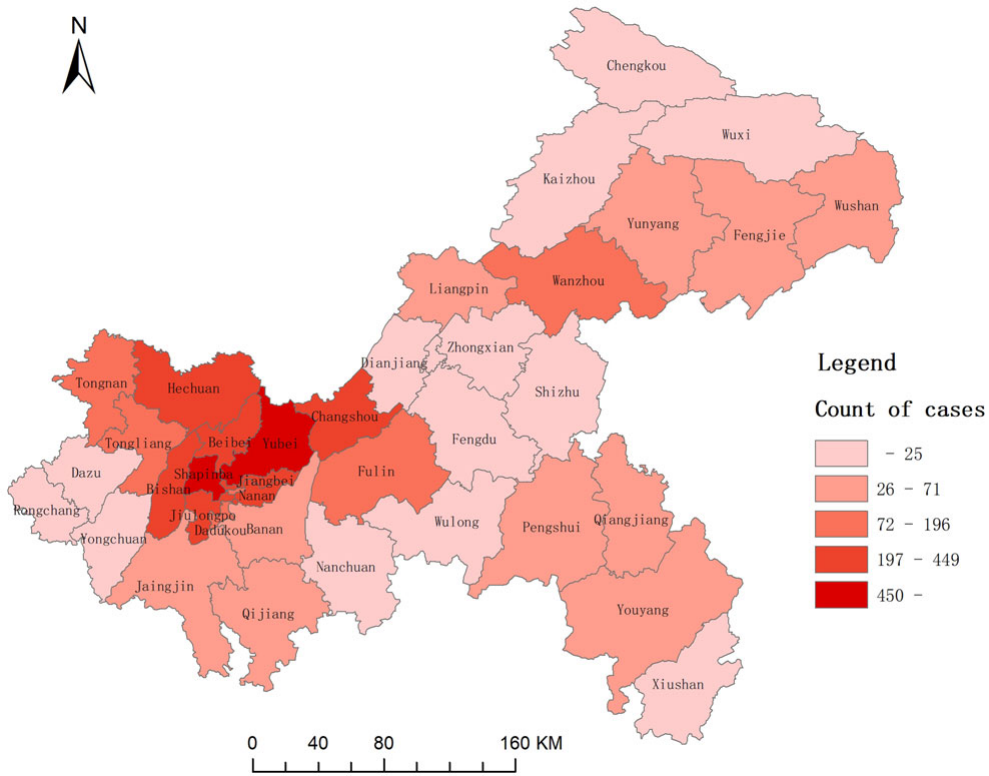
Spatial distribution of scarlet fever incidence in Chongqing, 2011–2019.

In general, the variation of geographical distribution of scarlet fever indicated that main urban districts had higher incidence than other regions. The top five districts reported most cases were Yubei (828 cases), Shapinba (599 cases), Jiulongpo (449 cases), Nanan (398 cases), Hechuan (350 cases). A total of 2624 scarlet fever cases were reported in these five districts, accounting for 51.72% of all cases (Fig. 5).

### SARIMA model

**Fig. 6.**
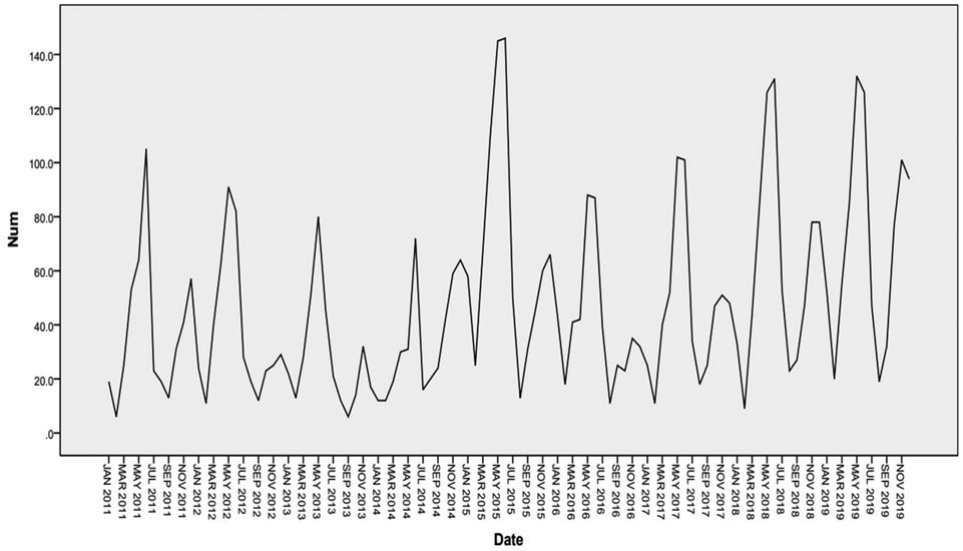
Sequence diagram of monthly scarlet fever incidence in Chongqing, 2011–2019.

**Fig. 7.**
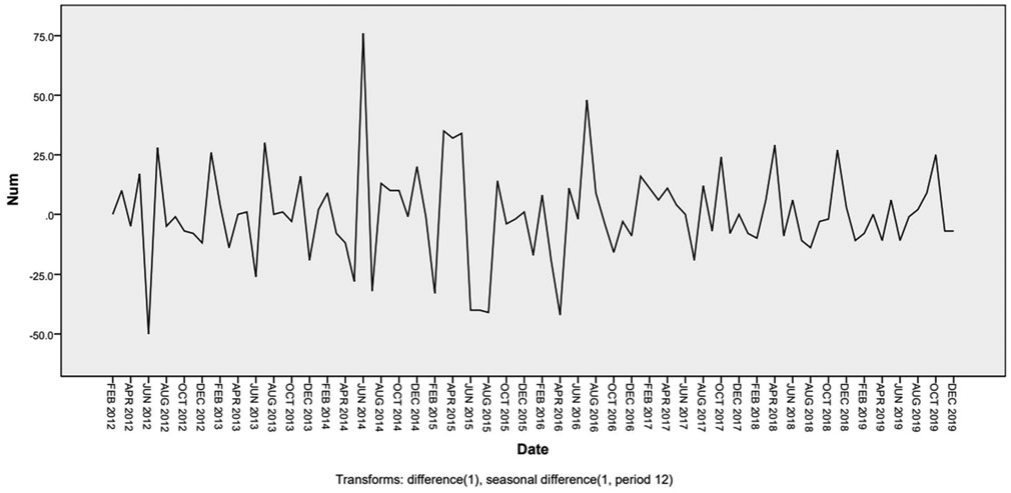
Sequence diagram of scarlet fever incidence after a first-order non-seasonal difference and first – order seasonal difference.

**Fig. 8.**
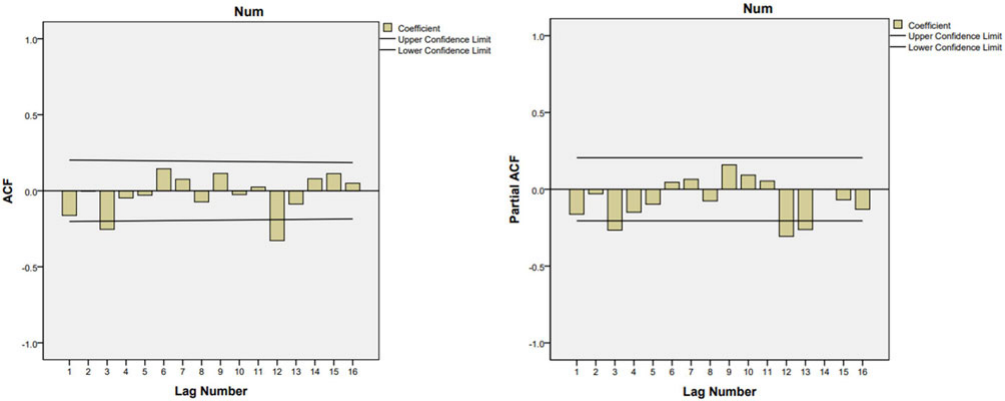
Autocorrelation function (ACF) and partial autocorrelation function (PACF) of monthly scarlet fever cases after a first-order non-seasonal difference and first – order seasonal difference.

As shown in Figure 6, the number of scarlet fever cases in Chongqing fluctuated with time in 2011–2019 years, which showing an obvious seasonal periodicity. We performed a first-order non-seasonal difference and a first-order seasonal difference with a period of 12 to eliminate the trends and seasonal effects, respectively. Figures 7 and 8 indicated that the differenced time series tend to be stationary.

**Fig. 9.**
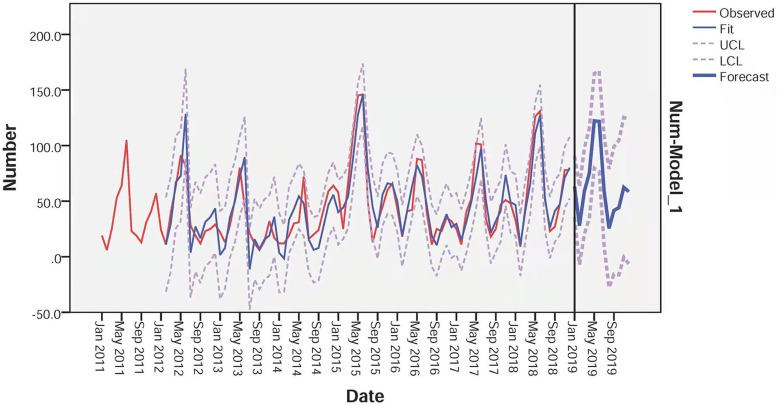
Observed and forecasted values of the SARIMA (3, 1, 3) (3, 1, 0)_12_ model (UCL, upper confidence limit; LCL, low confidence limit).

**Fig. 10.**
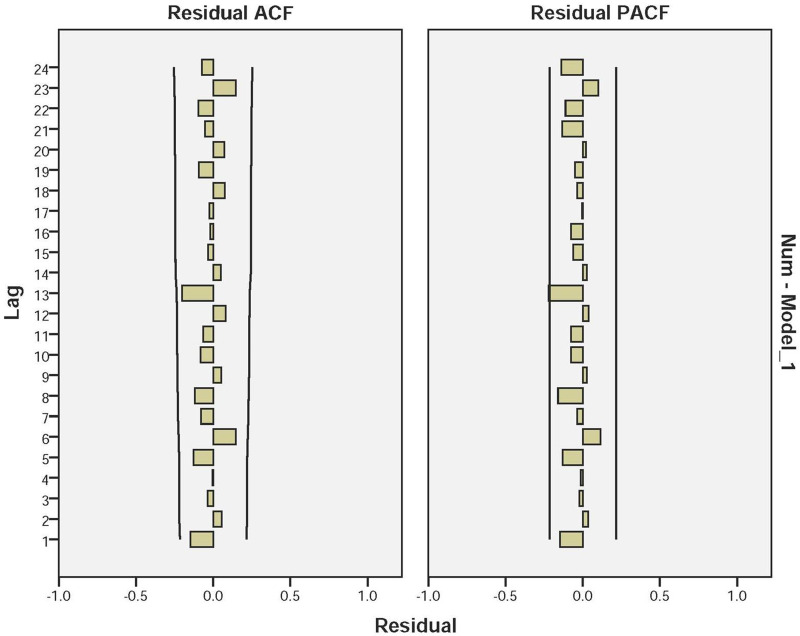
Autocorrelation function (ACF) and partial autocorrelation function (PACF) of the residuals series of the SARIMA (3, 1, 3) (3, 1, 0)_12_ model.

According to the characteristics of ACF and PACF distribution (Fig. 8), we selected four models provisionally, and excluded the models in which the residuals were not likely to be white noise. Based on the results of the goodness-of-fit test statistics, we confirmed the optimal SARIMA (3, 1, 3) (3, 1, 0) _12_ model, which had the lowest BIC (BIC = 6.054), lowest RMSE (RMSE = 15.81) and relatively high *R*^2^ (*R*^2^ = 0.789). Estimation of the SARIMA model parameters and the testing results were presented in Table 2 and S1 in Supplementary material. Figure 9 showed that the observed values and fitted values of the model matched well. Furthermore, the ACF and PACF of the residuals of SARIMA (3, 1, 3) (3, 1, 0)_12_ fell within the random confidence interval, indicating that the residuals did not deviate from a zero-mean white noise process (Fig. 10). Finally, the results of the Ljung-Box *Q* test (*Q* = 14.663, *P* = 0.101) for the model suggest that the residual series was a white noise sequence. The results of Goodness-of-fit test demonstrated that the performance of the fitted SARIMA (3, 1, 3) (3, 1, 0)_12_ model was reasonably well.

**Table 2.** Goodness of fits for the SARIMA models corresponding to different choices of *p*, *q* and *P*, *Q* which had passed the white noise test

Model	BIC	*R*^2^	RMSE	Ljung-Box *Q* statistics	Sig.
SARIMA (3, 1, 3) (3, 1, 0)_12_	6.054	0.789	15.81	14.663	0.101
SARIMA (3, 1, 3) (3, 1, 1)_12_	6.116	0.79	15.883	12.597	0.126
SARIMA (3, 1, 3) (3, 1, 2)_12_	6.18	0.79	15.971	11.108	0.134
SARIMA (3, 1, 3) (3, 1, 3)_12_	6.244	0.791	16.056	11.759	0.068

**Fig. 11.**
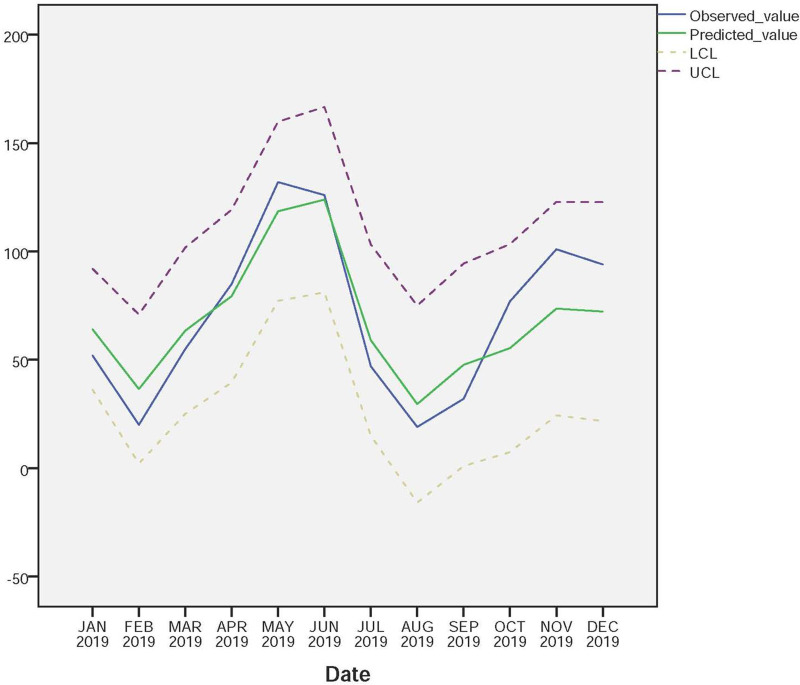
Sequence diagram of observed and predicted values of scarlet fever in 2019 (UCL, upper confidence limit; LCL, low confidence limit).

Finally, the SARIMA (3, 1, 3) (3, 1, 0) _12_ model was applied to forecast the monthly incidences of scarlet fever cases for 12 months from January to December in 2019. Figure 11 showed the comparison of observed and forecasted scarlet fever cases by the model, and the observed cases were within 95% confidence interval (CI) of the forecasted values. Table 3 showed the values of observed and predicted in 2019. The RMSE and MAPE between the predicted and actual values were 19.40, and 0.25 respectively, which verified the feasibility and effectiveness of the SARIMA (3, 1, 3) (3, 1, 0)_12_ model.

**Table 3.** Comparison of observed and forecasted scarlet fever from January to December in 2019 by the SARIMA (3, 1, 3) (3, 1, 0)_12_ model

Month	January	February	March	April	May	June	July	August	September	October	November	December
Actual	52	20	55	85	132	126	47	19	32	77	101	94
Predicted	63.07	28.02	59.02	75.18	122.25	121.78	59.21	25.56	41.69	44.09	62.40	58.70

## Discussion

Over the past decade, the incidence of scarlet fever has an anomalous increase in some Asian and European countries and regions, and this trend had exerted a marked influence on Chinese population since 2011 [19]. It is necessary to fully understand the epidemiological characteristics and predict the incidence of scarlet fever to provide information and evidence for its prevention and control.

Even though strategies and measurements, such as symptoms surveillance in the gate of kindergartens and primary schools, timely isolation of cases and daily disinfecting of environment, were brought up for intervening the transmission of scarlet fever, the incidence of scarlet fever increased sharply from 2004 to 2019 in China and other countries [6, 20, 21]. Previous studies indicated that meteorological factors and air pollution factors may lead to an increase in scarlet fever incidence, such as ambient particulate matter, nitrogen dioxide, temperature and relative humidity were significantly associated with scarlet fever incidence [22–24]. Moreover, some studies suggested that the rapid increase trend may be associated by the overuse of antibiotics, which would bring mutation and drug resistance to *Streptococcal* species [25]. However, the specific mechanism was remained unclear, and more research studies are needed in the future.

The time series of scarlet fever exhibited semiannual peaks in Chongqing, with a large peak during April to June and a small peak during October to December. The two peaks were coincided with local school semesters, which was similar to other provinces in China [23, 26]. Therefore, it is crucial to enhance scarlet fever control and prevention measures in schools.

The incidence of scarlet fever was higher in boys, with a male-to-female ratio of 1.44. This observation was similar to the findings in Korea [27], mainland China [5, 28]. Consistent with previous studies, males were more frequently infected by scarlet fever than females, this phenomenon may be related to some factors including male susceptibility at the host genetic level, host immune status, behaviour pattern, and it may also be due to reporting bias. The incidence of scarlet fever was highest among 3–9 years of age during 2011–2019. A study in Zhejiang, China, also reported similar finding from 2004 to 2018 [20]. In addition, several studies in Hong Kong [6] and England [5] indicated that the most prevalent ages of scarlet fever were 3–5, and 3–7, respectively. Therefore, kindergartens and primary schools are suggested to be the focus of monitoring and control of scarlet fever. It may be helpful to strengthen the monitoring and reporting of scarlet fever cases, so as to achieve early detection, early diagnosis and early isolation.

In this study, we found that the SARIMA (3, 1, 3) (3, 1, 0)_12_ model was the best-fitted mathematical model for forecasting monthly scarlet fever incidence based on the surveillance data from January 2011 to December 2018 in Chongqing, China. The model was then used to predict the incidence of scarlet fever in Chongqing in 2019. The results showed that the actual monthly incidence was within 95% CI of the predicted values, and the RMSE and MAPE between the predicted values and the actual values was 19.40 and 0.25 respectively, indicating that the model has reasonable accuracy in predicting the incidence of scarlet fever in Chongqing. Compared with the actual values, there had slight difference in predicted value in 2019, but the change trends of the two were basically the same. Due to the incidence of infectious disease was affected by complex factors, the time series model cannot predict it absolutely accurately, but the predicted value of the model can still accurately estimate the possible increase in the future.

The SARIMA model requires at least 7 or 8 seasonal cycles to estimate seasonal parameters [29]. We used nine seasonal cycles of incidence of scarlet fever, which meet the requirements of applying the SARIMA model. However, the epidemic of scarlet fever was affected by climates, environments and other factors [22–24], and the change of any factor may lead to variety of the epidemic law of the disease. Therefore, the single constructed prediction model cannot be used as a permanent prediction tool. The prediction model should be adjusted according to the changes of the actual situation. Besides, further studies should explore variables that mainly affect the incidence of scarlet fever, including environmental factors, host factors and pathogen factors, and then incorporate them into the prediction model [30].

To the best of our knowledge, this is the most comprehensive study of scarlet fever up to now in Chongqing, China. The findings can be helpful for the control and prevention of scarlet fever epidemics in this area.

Several limitations of this study should be mentioned when interpreting the predicting results. First, the data were obtained from NDRIS, and the subclinical or asymptomatic cases of scarlet fever without seeking medical consultation were not reported in the system, therefore the reported data may underestimate the incidence. Second, the SARIMA model was only applicable to short-term prediction, and the model needs to be dynamically updated with new data to ensure the accuracy and stability of the prediction. Third, meteorological and environments factors such as temperature, rainfall, and PM_10_ may affect scarlet fever epidemics, but we were unable to incorporate these data into our study. Finally, we did not have data for *emm* gene typing of group A *streptococcus* in Chongqing.

In conclusion, the study provides key epidemic characteristics of scarlet fever in Chongqing, China. The SARIMA model can effectively predict the short-term scarlet fever incidence. The prediction model could be helpful in developing timely disease control and prevention strategies for scarlet fever in Chongqing, such as properly allocating health resources.

## Data Availability

The datasets used and analysed during the current study is available from the corresponding author Li Qi (E-mail: qili19812012@126.com) on reasonable request.

## Supplementary material

For supplementary material accompanying this paper visit http://doi.org/10.1017/S0950268822000693. click here to view supplementary material
