# Supplementary material for: Forecasting the monthly incidence of scarlet fever in Chongqing, China using the SARIMA model
Source: Epidemiol Infect. 2022 Apr 21;150:e90. doi: 10.1017/S0950268822000693 (PMC9102071; doi:10.1017/S0950268822000693)
Supplement: Supplementary file 1 [file S0950268822000693sup001.docx]

*Epidemiology and Infection*

**Forecasting the monthly incidence of scarlet fever in Chongqing, China using the SARIMA model**

**Authors**

W. W. Wu^1,*^, Q. Li^1,*^, D. C. Tian^2,*^, H. Zhao^1^, Y. Xia^1^, Y. Xiong^1^, K. Su^1^, W. G. Tang^1^, X. Chen^1^, J. Wang^1^, L. Qi^1,^

Supplementary Material

**Supplementary Table S1** Parameter estimation for plausible SARIMA models

|  |  | AR | | MA | | Seasonal AR | | Seasonal MA | |
| --- | --- | --- | --- | --- | --- | --- | --- | --- | --- |
|  |  | *B* | *P* | *B* | *P* | *B* | *P* | *B* | *P* |
| SARIMA(3,1,3)(3,1,0)_12_ | Lag 1 | 0.224 | 0.203 | 0.431 | 0.2 | -0.832 | <0.001 | - | - |
|  | Lag 2 | 0.477 | 0.001 | 0.64 | 0.045 | -0.797 | <0.001 | - | - |
|  | Lag 3 | -0.744 | <0.001 | -0.752 | <0.001 | -0.303 | 0.034 | - | - |
| SARIMA(3,1,3)(3,1,1)_12_ | Lag 1 | 0.296 | <0.001 | 0.542 | 0.001 | 0.977 | 0.886 | - | - |
|  | Lag 2 | 0.487 | <0.001 | 0.566 | 0.008 | - | - | - | - |
|  | Lag 3 | -0.808 | <0.001 | -0.811 | <0.001 | - | - | - | - |
| SARIMA(3,1,3)(3,1,2)_12_ | Lag 1 | 0.34 | 0.175 | 0.629 | <0.001 | -0.89 | 0.391 | -0.019 | 1 |
|  | Lag 2 | 0.469 | 0.112 | 0.459 | 0.007 | -0.313 | 0.508 | 0.977 | 0.989 |
|  | Lag 3 | -0.842 | <0.001 | -0.809 | <0.001 | -0.05 | 0.92 | - | - |
| SARIMA(3,1,3)(3,1,3)_12_ | Lag 1 | 0.439 | 0.051 | 0.74 | 0.003 | -0.828 | 0.973 | -0.038 | 0.999 |
|  | Lag 2 | 0.36 | 0.188 | 0.298 | 0.411 | -0.242 | 0.99 | 0.914 | 0.968 |
|  | Lag 3 | -0.787 | <0.001 | -0.731 | 0.002 | -0.015 | 0.998 | -0.034 | 0.999 |

***Abbreviation*:** AR= Autoregressive, MA= moving average
